# Determinants of participation and quality of life of young adults with cerebral palsy: longitudinal approach and comparison with the general population – SPARCLE 3 study protocol

**DOI:** 10.1186/s12883-021-02263-z

**Published:** 2021-06-30

**Authors:** Catherine Arnaud, Carine Duffaut, Jérôme Fauconnier, Silke Schmidt, Kate Himmelmann, Marco Marcelli, Lindsay Pennington, Joaquim Alvarelhão, Chirine Cytera, Marion Rapp, Virginie Ehlinger, Ute Thyen

**Affiliations:** 1 UMR 1027 Inserm, Toulouse3 University, team Sphere, Hôpital Paule de Viguier, 330 Avenue de Grande Bretagne, TSA 70034, F-31059 Toulouse, France; 2 Clinical Epidemiology Unit, University Hospital, F-31059 Toulouse, France; 3 Laboratoire TIMC-IMAG Equipe ThEMAS, Grenoble Alpes University, Pavillon Taillefer CHU Grenoble CS10217, F-338043 Grenoble, France; 4 University of Greifswald, Institute of Psychology, Robert-Blum-Str. 13, 17489 Greifswald, Germany; 5 Institute of Clinical Sciences, University of Gothenburg, Gothenburg, Sweden; 6 Azienda Sanitaria Locale Viterbo, Child and Adolescent Neuropsychiatric Unit – Adult Disability Unit, Via Enrico Fermi 15, 01100 Viterbo, Italy; 7 Population Health Sciences Institute, Newcastle University, Newcastle upon Tyne, UK; 8 School of Health Sciences, University of Aveiro, Campo Universitário de Santiago, Aveiro, Portugal; 9 Department of Pediatric and Adolescent Medicine, Universität zu Lübeck, Ratzeburger Allee 160, 23538 Lübeck, Germany

**Keywords:** Cerebral palsy, Participation, Quality of life, Health care, Employment, Adulthood

## Abstract

**Background:**

Effective inclusion in society for young people with disabilities is increasingly seen as generating opportunities for self-development, and improving well-being. However, significant barriers remain in the vast majority of activities meaningful for young adults. Research argues that various personal (disabilities, health) and environmental (access to the resources needed, accessible environment, discrimination, lack of personal economic independence) factors contribute to limited participation. However, previous studies conducted in young people with cerebral palsy (CP) mainly investigated the transition period to adulthood, and did not fully consider the whole range of impairment severity profiles or environmental barriers. In this study, we will use the follow-up of the SPARCLE cohort and a comparison group from the general population (1) to investigate the impact of the environment on participation and quality of life of young adults with CP, (2) to determine predictors of a successful young adulthood in educational, professional, health and social fields, (3) to compare quality of life and frequency of participation in social, work and recreational activities with the general population, (4) to document on participation and quality of life in those with severe disabilities.

**Methods:**

The SPARCLE3 study has a combined longitudinal and cross-sectional design. Young adults with CP aged 22 to 27 years in 6 European regions previously enrolled in the SPARCLE cohort or newly recruited will be invited to self-complete a comprehensive set of questionnaires exploring participation (daily life and discretionary activities), health-related quality of life, body function, personal factors (health, personal resources), and contextual factors (availability of needed environmental items, family environment, services provision) during home visits supervised by trained researchers. Proxy-reports or adapted questionnaires will be used for those with the most severe impairments. The recruitment of a large group from the general population (online survey) will enable to identify life areas where the discrepancies between young people with CP and their able-bodied peers are the most significant.

**Discussion:**

This study will help identify to what extent disabilities and barriers in environment negatively affect participation and quality of life, and how previous valued experiences during childhood or adolescence might modulate these effects.

## Background

Young adulthood is an important transitional life phase, particularly challenging for those living with disabilities. Despite they are nowadays enjoying better health care, new communication technologies, and benefiting from societal attitudes allowing inclusion in all areas of living [1], they continue to face numerous barriers in their daily lives. Research indicated that amongst others, psychological distress, fragmented social interactions, poor accessibility, discrimination linked to disability, and lack of personal economic independence contribute to limited involvement in life situations [2–4] that can extend across adulthood. Another area of concern is their ability to access needed resources that may improve autonomy, self-efficacy and health [5]. Differently from their able-bodied peers who use health care infrequently in young adulthood, those with childhood-onset disabilities such as cerebral palsy (CP) remain dependent on the health care system [6]. Notwithstanding that they encounter health challenges such as deteriorating mobility, high rates of pain and fatigue, mental health problems [7–11], they may decrease its utilization, in part because the loss of individualized supports and services can be difficult to overcome and because the transition to adult-orientated services is poor [12–14].

Previous research reported that meaningful contribution and integration in the community are beneficial and largely contribute to personal well-being and quality of life (QoL) [15]. However, knowledge on QoL in young adults with CP is limited and results inconsistent when comparing to general population of same age [8, 16, 17]. Overall, presence of disability is not automatically associated with poor QoL [8, 17], but those with more severe impairments, notably severe cognitive impairments, consistently reported low levels of QoL [16]. With respects to participation, studies mainly conducted during childhood or adolescence showed that social participation is highly impacted by disability and overall lower than that reported by able-bodied peers [18, 19]. The literature also suggested that age might be a determinant of participation [20] and valued previous experiences of participation an anchor for future participation [21]. However, there is increasing evidence that social participation of young adults with CP remains uncertain and highly dependent on parental support [4, 22, 23]. As adults, they still have few opportunities for personal development [24] and poor involvement in meaningful activities such as employment [25, 26]. Thus, mainstream schooling and inclusive education [27, 28] which are recognised as instrumental in fully participating in society do not guarantee effective access to postsecondary education, labour market, paid work, or sustainable appropriately compensated employment [20, 29–32].

Studies are still lacking regarding young persons with severe physical disabilities, and multiple severe co-morbid conditions (intellectual and communication disorders) [33]. There are inherent challenges to evaluate their QoL, one of them being the uncertainty as to whether proxy-ranking is able to provide a meaningful appraisal [34]. We still need to gain a deeper understanding of the factors that contribute to their well-being [35]. The meaning of participation in this population is also of complex nature and research is scarce to understand how the needs for personal development, social belonging, and choice of activities are expressed [36]. While the needs seem to be similar to that of their less severely impaired or able-bodied peers, mediators of participation are different. Individual factors (health, function, time and effort), family factors (good health, motivation of the parents), financial and organizational resources that respond to the needs, accessible environments, adult assistance to care for and supportive networks shape their participation in recreational activities and their social roles [37].

In line with the WHO conceptual model where the disabled condition results from the inadequacy of the environment to the person [38], the relationships between environmental factors, participation and QoL have been extensively studied by the European network SPARCLE (Study of PARticipation in Children with cerebral palsy Living in Europe) [39, 40]. Policies and practices in physical environment, support and care services, education as well as access to the needed environment [41] showed substantial differences across participating countries that brought an enhanced knowledge on to what extent a non-supportive environment affect participation and QoL. The targeted population was children (first wave) and adolescents (second wave of the study) with CP. CP is the leading cause of early-onset physical disability occurring in about 2 in 1000 livebirths [42–44]. It results from non-progressive lesions to the developing brain of the foetus or new-born [45] leading to a set of permanent disorders affecting movement and posture. In addition to mobility issues, persons with CP may experience a wide range of difficulties (intellectual disability, epilepsy, communication difficulty, visual or hearing impairment, secondary musculoskeletal problems, pain, …) resulting in a diversity of disability profiles [46].

Overall, there is a lack of studies which explore how personal and environmental factors influence participation and QoL in young adults with CP across the whole range of severity profiles both in a cross sectional approach (comparison with the general population) and from a life-course perspective (predictors of participation and QoL) [47, 48]. Also, most studies were conducted in single countries, with a limitation being that environmental barriers were not fully considered. As such, the study aims are fourfold. First, to investigate the interactions between personal and environmental factors and their impact on participation and QoL in young adults with CP. Second, to characterize the trajectories from childhood and determine the predictors of a successful young adulthood in the educational, professional, health and social fields. Third, to compare the frequency of participation in social, work and recreational activities, as well as QoL as reported by young adults with CP to the general population. Fourth, to report on participation and QoL in those with severe disabilities (physical and additional impairments) by using adapted materials to obtain self-assessments when possible, or proxy-reports.

## Methods/design

### Design and setting

**Fig. 1:**
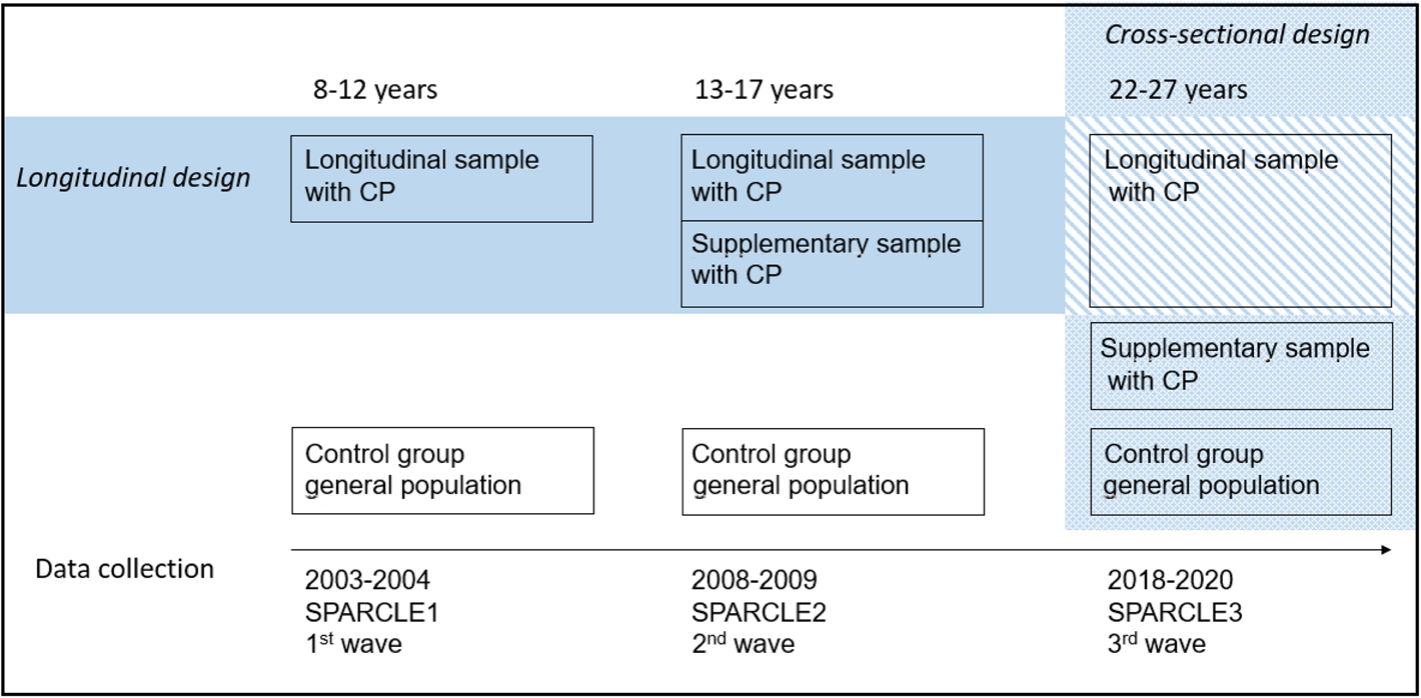
Study design

SPARCLE3 is a multicentre European observational population-based study combining the follow-up of the SPARCLE cohort to young adulthood (22–27 years) and a cross-sectional part allowing the recruitment of a larger sample of young people with CP and a representative sample of the general population of same age. The general design is showed in Fig. 1. The study will be implemented in five of the nine European regions originally investigated: South West and South East France (Haute-Garonne and Isère counties respectively), North West Germany, Western Sweden (region of Goteborg), Central Italy (Viterbo area). The region of Porto (Portugal) will be added to cross-sectional part.

### Study participants

The eligible population for SPARCLE3 consisted of young adults born between 31/07/1991 and 01/04/1997 with confirmed diagnosis of CP as defined by the SCPE [49]. Overall, those who participated in at least one of the two first waves of the SPARCLE study (SPARCLE1 in childhood [39] and/or SPARCLE2 in adolescence [40]) will be eligible to the *Longitudinal CP sample.* Initially, the SPARCLE cohort randomly sampled children with CP from population-based registries thus selecting children across the whole range of severities. In addition, children with the most severe impairments were over-represented (random sampling method stratified on walking ability level). In one region, children with CP of similar age, gender, and levels of impairment were recruited from multiple sources. In total, 387 children with CP aged 8 to 12 years living in the five regions participating in SPARCLE3 were enrolled in the first wave. Among them, 278 (72%) agreed to participate in the second wave of the study when they were 13 to 17 years old. An additional sample of 29 adolescents was added in the second wave in order to maintain statistical power for cross-sectional analyses and follow-up to adulthood. Thus, 416 young adults with CP will be eligible to the *Longitudinal CP sample*. We will use all the available information to trace the cohort.

A supplementary sample of young adults with CP aged 22 to 27 years at time of interview, who have not participated in SPARCLE 1 nor SPARCLE 2, will be recruited from multiple sources (paediatric/young people rehabilitation centres, paediatric hospitals, institutions for people with intellectual deficiencies) in North Germany, and using the database of Cerebral Palsy Rehabilitation Centre of Porto (CR-APPC) in Portugal in order to compensate for drop-outs and have sufficient power for sub-groups analyses. In both regions, articles about the study will be published in magazines and newsletters for people with disabilities and for physicians, and project flyers will be distributed to organisations engaged in the target group, physician offices, therapists and clinics specialised in the treatment of young people with CP (Germany) or at Web forums and Facebook groups of the Cerebral Palsy Association of Porto (Portugal). Similar procedures to contact the participants will be used. Professionals will introduce the study to potential participants or their representatives by phone or mail. Patients willing to participate will receive information material. An additional contact will confirm the participation before the consent is signed and the interview schedules. In the other regions, persons with CP recorded in CP registries but not earlier randomized in the SPARCLE study will also be considered for inclusion in the *Supplementary CP sample*. Representativeness of CP samples will be assessed against expected values.

A large comparison group from the general population in the different countries will be enrolled via a panellist. It will include males and females (in a 50%/50% ratio) aged 19–29 years living in the participating countries. All residents in the participating countries aged 19–29 represent the respective target populations. The online participants will be recruited randomly from all panellists involved via an online access panel according to the specifications of the target matrix. The panellists will also be responsible for the management of the invitations and recruitments. Each participant will receive a personal ID via their invitation link. After completing the survey, this ID will be returning to the panel via a redirect link. The panellists will use screen-out links to track participants who do not fit into the intended age group (exclusion criterion). Prospective participants will be requested to submit their informed consent by registering at the panel provider and consent to get contacted for study participation requests. The survey will be strictly anonymous, without access to personal contact details by the members of the SPARCLE group. Computer-assisted web interviewing will be used for this survey study. Participants will receive an incentive for completing the survey. Given the randomized nature of the recruitment procedure and the predefined quotes of the target matrix for matching the patterning of the sociodemographic characteristics of the general population, the survey design is suitable to minimize potential incentive-caused bias related to the selection, enrolment and representation of participants.

Table 1 reports figures of eligible samples and anticipated recruitments, stratified by group and region.

**Table 1:** Expected recruitments by group (young adults with cerebral palsy and general population) and region

Regions	Young adults with CP^a^			General population
	Longitudinal sample		Supplementary sample	
	Eligible from previous SPARCLE waves	Expected recruitment^b^	Expected recruitment	Expected recruitment
France, 2 centres	162	97	0	1000
Germany	85	51	55 to 65	1000
Sweden	83	49	0	1000
Italy	86	51	0	500
Portugal			55 to 65	500
Total	416	248	160	4000

^a^ *CP* Cerebral palsy

^b^ Anticipated participation rate: 60%

### Ethics approval and consent to participate

Relevant ethics and regulatory authorizations were sought in each country and the study fully approved. All young people with CP or their legal representatives will give informed consent to participate, or non-opposition where appropriate. Participants from the general population will be informed at the beginning of the online survey and will give their informed consent before being allowed to start the survey. The online survey will be strictly anonym without any possibility to access to the contact data of participants.

### Data collection procedures

All participants in the SPARCLE cohort (*Longitudinal CP sample*) previously gave their permission to be contacted again for a further study. Prior to any investigation, the vital status of the participants will be checked. After a phone contact, written information about the objectives of the study and the activities for participants will be sent. There will be at least 7 days between arranging the appointment for a home visit and completion of questionnaires after the final consent is obtained. Pretesting the questionnaires, we estimated that the participation in the study will take between 2 to 6 hours, which may spread over 1 or 2 days according to participant’s health. Persons who moved out of the catchment area will be contacted and possibly included (home visit if feasible, otherwise telephone interview). For new participants (*Supplementary CP sample*), a health professional such as a neurologist or a general practitioner will approach the person or their legal representative. Apart from this, similar procedures for home visits will be applied.

Information will be collected by trained researchers to ensure consistency across regions. Questionnaires will be administered in a logical flow and fixed order, and self-completed whenever possible. For persons with motor or communication difficulties or unclear speech, the research assistant will explain, answer queries, and assist if needed. Picture versions of some instruments or answering scales will be used at the research assistant’s discretion. In case self-assessment will not be possible even with help, the questionnaires will be filled out by the personal assistant or any individual (including parents) who knows the young adult very well (meaning that they are significantly involved in their daily life). For the general population, information will be collected using computer-mediated communication. The completion duration will be 10 to 20 min.

### Measurements

All data collected refer to the WHO International Classification of Functioning, disability and health (ICF) [38], using cross-cultural and validated instruments where possible, and adding questions developed in the framework of the project when necessary. The chosen instruments and their characteristics, as well as the populations investigated are summarized Table 2.

**Table 2:** Instruments to be used in SPARCLE3

ICF construct	Instrument	Captures	Domains	Number of Items	Response scale, Recall period	Populations investigated	Used in previous SPARCLE waves	References
	WHO-QOL BREF Disabilty module Picture version Talking mats	Health related Quality of life	•Overall Quality of Life and General Health •Physical Health •Psychological •Social relationships •Environment	26	5-point Likert scale past 2 weeks	• Longitudinal CP sample • Supplementary CP sample • General population	No	Skevington SM, et al. (2004). Quality of Life Research; 13(2), 299–310 (ref [50]) The WHOQOL Group. (1998) Psychol Med, 28(3), 551–558. (ref [51])
	Kidscreen 10 Index	Health related Quality of Life 8–18 years	Global Health related QoL index	10	5-point Likert scale	Longitudinal CP sample	SPARCLE 1 SPARCLE 2	Ravens-Sieberer U, et al. (2010) Qual Life Res;19(10):1487–500. (ref [52])
Participation	WHODAS 2.0 WHO Disability Assessment Schedule	Activities (5 dimensions) Participation	• Cognition • Mobility • Self-Care • Getting along • Life activities • Participation	12	5-point Likert scale Past 30 days	• Longitudinal CP sample • Supplementary CP sample	No	Ustun TB et al. Manual for WHO Disability Assessment Schedule – WHODAS 2.0 (WHO 2010) (Ref [53])
	QYPP-YA Questionnaire of Young People Participation (version adapted for young adults from the original QYPP)	Frequency of discretionary participation	• Autonomy • Getting on with other people • Community recreation • Finances • Work/education • Sexuality • Media	22	5-point Likert scale	• Longitudinal CP sample • Supplementary CP sample • General population	SPARCLE1 (FPQ) SPARCLE2 (QYPP version adapted for adolescents)	(QYPP) Tuffrey C, et al. (2013), Child Care Health Dev; 39(4):500–11 (ref [54])
Environmental factors	EAEQ European Adult Environment Questionnaire (version adapted from the original ECEQ)	Availability of needed environmental items	• Home Environment •Work/college/university/day placement • Public places •Transport • Finance • Services	61	•Not needed•Needed and available •Needed and not available	• Longitudinal CP sample • Supplementary CP sample	SPARCLE1 (original version for children) SPARCLE2 (original version for children)	Dickinson HO et al. (2011) Disabil Rehabil; 33(1):36–50 (ref [55])
	YHC-SUN Youth Health Care – Satisfaction, Utilization and Needs	Service provision	• Access • Perceived satisfaction with health care	30	5-point Likert scale	• Longitudinal CP sample • Supplementary CP sample	no	Schmidt et al. BMC Health Services Research (2016) 16:189 (ref [56]) Adapted from the long form
	Transition competence scale	Medical care	Health care competence	3	4-point Likert scale	• Longitudinal CP sample • Supplementary CP sample	no	Hermann-Garitz C, et al. (2017) Gesundheitswesen; 79(06):491–6 (ref [57])
		Socio economic background of the family		2		• Longitudinal CP sample • Supplementary CP sample	SPARCLE1 SPARCLE2	
Body function and structure	GMFCS and BFMF (adult version) Washington Group Short Set of Disability Questions	Type and severity of impairments	• type of CP (SCPE) • motor function • seizures • cognitive impairment • vision, hearing • pain (frequency, amount, location) • general health	14		• Longitudinal CP sample • Supplementary CP sample	SPARCLE1 SPARCLE2	Mc Cormick A et al. (2007) Dev Med Child Neurol; 49(4):265_9 (ref [58] Elvrum A-KG (2017); Phys Occup Ter Pediatr; 37(3): 239–51 (ref [59]) Washington group on Disability statistics (ref [60])
	EDACS (Eating and Drinking Ability Classification System) Feeding scale	Eating and drinking abilities		1	5 levels of severity	• Longitudinal CP sample • Supplementary CP sample	no	EDACS [internet] (ref [61]) Sellers D et al. (2014); 56(3): 245–51 (ref [62])
	VSC (Viking Speech Scale)	Speech abilities		1	4 levels of severity	• Longitudinal CP sample • Supplementary CP sample	no	Pennington L et al. (2013) Res Dev Disabil; 34(10):3202–10 (ref [63]
	CFCS (Communication Function Classification System)	Communication abilities		1	5 levels of severity	• Longitudinal CP sample • Supplementary CP sample	no	Hidecker MJC et al. (2011) Dev Med Child Neurol;53(8):704–10 (ref [64])
	SMIS (St. Mark’s hospital Incontinence Score)	Bowel dysfunction	• frequency • strategic adaptations	7	5-point Likert scale Y/N questions	• Longitudinal CP sample • Supplementary CP sample	no	Vaizey CJ et al. (1999) Gut; 44(1):77–80 (ref [65])
	ISI (Incontinence severity Index) Revised Sandvik Scale	Bladder dysfunction	• frequency • amount of leakage.	2	4 levels of severity	• Longitudinal CP sample • Supplementary CP sample		Sandvik H et al. (2006) Int Urogynecol J Pelvic Floor Dysfunct; 17(5): 520–4 (ref [66])
	BaSIQS Basic Scale on Insomnia complaints and Quality of Sleep	Sleep Problems	• sleep quality • insomnia complaints	7	Last month	•Longitudinal CP sample •Supplementary CP sample •General population	no	Allen Gomes A et al. (2015) Chronobiol Int; 32(3):428–40 (ref [67])
	GAD 7 Generalized Anxiety Disorder	Anxiety	frequency of anxious symptoms	7	4-point Likert scale Last 2 weeks 4 levels of severity	•Longitudinal CP sample •Supplementary CP sample •General population	no	Spitzer RL et al. (2006) Arch Intern Med; 166(10):1092–7 (ref [68])
	PHQ 9 Patient Health Questionnaire	Depression	degree of depression severity	9	4-point Likert scale Last 2 weeks 5 levels of severity	•Longitudinal CP sample •Supplementary CP sample •General population	no	Kroenke K et al. (2001)J Gen Intern Med; 16(9): 606–13 (ref [69])
	GSE General Self Efficacy scale	Self-efficacy	–	10	4-point Likert scale Total score	•Longitudinal CP sample •Supplementary CP sample •General population	no	Schwarzer et al. (1995) (ref [70])
Personal factors	Social determinants	Current status of personal social determinants	• housing •lifestyle • educational level (3 levels) • current activity • income • children • social and familial relationships • spirituality	18		•Longitudinal CP sample •Supplementary CP sample •General population	SPARCLE1 SPARCLE2 (some indicators + new items)	Some questions issued from the European Social Survey (ref [71])
	Risky behaviours		• smoking behaviour • alcohol drinking behaviour	5		•Longitudinal CP sample •Supplementary CP sample •General population	no	

Briefly, QoL will be assessed using the WHOQOL-BREF [50, 51]. Because reference data is difficult to obtain for the studied age range, the instrument will be completed by the general population as well. The disability answering scale (disability module [72]) will be used when necessary. A picture version (adapted Talking Mats version [73]) will be used optionally to enable those with the most severe disabilities to self-report, in addition to proxy-reports. Although not developed for the age range of the study participants, KIDSCREEN-10 index [52] will be used in the *Longitudinal CP sample* to allow direct comparisons across time. For participation, several instruments will be used. First, the WHO Disability Assessment Schedule (WHODAS) 2.0 instrument [53] will be filled in by young people with CP as a measure of difficulties in achieving activities in five dimensions, and participation in one. Second, we sought to measure participation as involvement in life situations from the perspective of participants with the frequency of attainment independent from the dimension of activities and functioning. Therefore, an instrument based on the previously validated Questionnaire of Young People’s Participation (QYPP [54];) was developed. Using group discussions, eight young adults with and without CP from northern Germany were asked about their perceptions of participation. In addition, five expert interviews were conducted with parents and professionals to indirectly include young adults with a more severe profile. The interviews were transcribed and analysed using qualitative content analysis. To fully reflect the areas identified (education and work, finances, leisure, social relationships, media, autonomy and mobility), 15 items were developed and added to the eight selected items of the original QYPP. Eleven cognitive interviews were then conducted to pilot the first version of the new instrument. They led to minor adaptations. The final version of QYPP-Young Adults (QYPP-YA) comprises 22 items measuring frequency of participation in selected areas of life. It was translated to the other languages according to international standards, and will be assessed in young people with CP and the general population.

We will use the European Adult Environment Questionnaire (EAEQ) to assess the availability of the needed physical, social and attitudinal environmental items, relevant and important to young people with CP. This instrument has been developed from the European Child Environment Questionnaire (ECEQ [55],), which was validated for children and young people with CP aged 8 to 17 years. Compared to the child version, the EAEQ includes additional questions about communication and technology in and outside the home, which were developed following interviews with young people with CP in the North of England and the carers of people with severe intellectual impairment. Content validity was assessed with a small number of English and Portuguese people (*n* = 40) through focus groups and cognitive interviewing. The validation study has explored the relevance of the new items, floor and ceilings effects, the instrument’s acceptability and ease to use including the duration of the questionnaire according to the severity profiles of the young people. Translations and back translations of the new items according to the international standards were performed. Other measures of the environment will capture family environment, access and perceived satisfaction with health care (using the YHC-SUN Youth Health Care, Satisfaction, Utilization and Needs, adapted from the long form [56]), and health-related transition competence (using selected items of the Transition competence scale [57]).

The ICF field “Body function and structure” that relates to the CP condition will be explored as follows: CP subtype, gross motor function [58] and two hands fine motor function [59], vision and hearing (Short Set of Questions on Disability, Washington Group [60]), seizures, eating and drinking ability [61, 62] with 3 complementary questions on feeding, speech ability [63], communication performance [64], bowel [65] and bladder [66] dysfunction, cognitive impairment, pain and discomfort, sleep problems [67]. Assessment of personal resources will include mental health [68, 69], self-efficacy [70], and risky behaviour.

With respect to personal factors, socioeconomic position and resources will be collected from all participants: level of educational attainment [74], place of residence, household, lifestyle. We will also ask for current status in terms of education, work, sources of income, and feeling about personal household’s present income [71].

### Data processing and data quality

All data of the CP sample required for the study will be entered using a standardized electronic Case Report Form on a dedicated database, with a secure interface reaching all conditions for maintaining the confidentiality and privacy of data. The database will be programmed to assist for accurate data entry (data dictionary, fields controls). Additional data validation will be done. At centre level, a random sample of 10% of cases will be audited against entered data by a staff not involved in the initial data entry. The acceptable error rate will be set at < 1% of the items. Further validations will be performed after exporting the data into a statistical package: extent and pattern of missing data, heterogeneity between centres, and potential for bias due to non-response. We will assess whether non-response is related to the severity of impairments, socioeconomic status of the individual, and whether any such association can explain differences in response rates between centres.

All data of the general population sample will be collected on line using appropriate software. After the targeted sample sizes have been reached, the survey data will be checked for sufficient quality according to various criteria, including interview duration, response variance of the matrices, and validity of the answers. Participants with an interview duration of less than half of the average time for completion will be referred to as “speeders”. The variances of the answers will be calculated for all matrices in the questionnaire (except for filter questions). Participants who will have an overall standard deviation below 0.5 will be assumed to show low response variance and referred to as “straight-liners”. Another criterion for invalid data will be “fake” responses to open questions. For this purpose, all open responses will be checked. If an answer does not correspond with the respective question or contain only a random sequence of letters, data of the respondent will be excluded.

### Power calculations

Considering that the cross sectional samples will comprise of 300 to 350 young adults with CP and a ratio of at least 1 in 10 for the CP sample as compared to that of the general population (Table 1), we will be able to document with over 80% power (type I error 0.05, bilateral) differences in participation between young adults with CP and those from the general population expressed as odds ratios (OR) (OR > 1 means that young people with CP participate less than the general population) as high as OR = 1.5 for activities achieved by 50% of the young adults with CP to 1.9 for less frequent activities (10% of the population with CP involved). Potentially eligible longitudinal CP sample consists of 416 young adults. Given an expected participation rate of 60%, we expect to enrol 245 persons of the SPARCLE cohort with CP.

### Statistical analyses

Psychometric analysis of the assessments will be applied, with special emphasis on revised, translated and newly developed measures. These analyses will include reliability, convergent and divergent validity and Differential Item Functionning across countries.

The study variables of main interest (participation, QoL, environmental factors) will be studied by domains. They correspond to unobserved or latent variables, manifested by responses to the items in the corresponding questionnaires. Measurement models will be specified and validated. For each domain, one model will be defined comprising a ‘measurement part’ that defines the latent constructs (sets of observed variables in accordance with Item Response Theory (IRT)), and a ‘structural part’ that hypothesises the links between these constructs in accordance with Structural Equation Modelling (SEM) methods. The structural part will specify the hypothesised links between the variables, both latent and observed. We will hypothesise that this domain in adulthood may be directly affected by the concurrent factors we will identify.

For the longitudinal analyses, we will hypothesise that each factor earlier in life could directly or indirectly (via its influence on mediating variables) affect a given domain in adulthood. The analysis will consider the same dimensions at the different periods as different variables, because of differences in questionnaires. The more parsimonious models will be taken in account. Sensitivity analysis will be performed to assess the possible effect of technical choices in parameters of SEM. Missing values will be imputed from predictors of drop-out using multiple imputation methods.

A conceptual overview on the association of QoL and well-being measures with the participation measure will be carried out. Therefore, we will explore a) the continuum of QoL and well-being measures, b) the conceptual inclusion of participation facets in QoL and well-being measures, and c) the impact of selective psychological comorbidity on participation. Using data collected in young adults with CP, we will investigate the comparability of the two WHOQOL-BREF versions for disabled persons vs. healthy persons [72], applying randomized assignment of different rating scale formats within the population based control group and applying item-response theory driven scaling and item parameter analysis.

## Discussion

This project is intended to document the impact of the physical, social, attitudinal, and health care services environment on participation and QoL in young adults with CP. We hypothesise that restricted participation of disabled people could be attenuated or even removed by changes in their environment with, for example, adaptations to their physical surroundings, the provision of equipment and an assumption of capacity by people in their community. The longitudinal perspective in addition with comparisons with the general population will improve our understanding on which factors of the environment have the higher impact on a beaming young adulthood.

We first designed a longitudinal approach with three time points allowing for intra-individual comparisons across time and the study of the shape of trajectories of interest. There are inherent conceptual and methodological issues in the assessment of change over time. One key issue was to determine the appropriate time point taking into account practical issues related to length of interval between previous and current waves (update of contacts, occurrence of migrations out of the area, attrition rate). In the SPARCLE3 study, we intended to explore the transition period of adolescence to young adulthood. However, we must expect a wide variation in developmental trajectories, also depending on the availability of resources and environmental, both physical and social, factors. Even if the concepts explored from childhood are similar, both the areas of interest and the representation of these concepts may change in the individual developmental course, which requires adaptation of questionnaires used in childhood or adolescence, or use of measurements developed for adults.

We also intend to compare the outcomes i.e. QoL, participation, psychological well-being, as well as the determinants (education, work, health and social fields, health care services) with the general population, and therefore selected instruments suitable for the general population. Online surveys, which are widely used for research, represent a potentially quick and affordable method of recruiting study participants from the general population. Of potential concern is the sample representativeness. We will compare overall figures to those obtained from other representative surveys whenever possible. Comparative analyses will help identify in which areas the opportunities for general health, psychological well-being, education, employment, independent living, social relations, and participation are the most afflicted, and determine the impact of environmental factors including access to health care. Comparisons between regions will identify if variations exist in patterns due to different approaches to organize services (notably the health care system) and support. Finally, our study will provide policy-makers, health professionals, and families (parents, siblings) with rigorous data collected in various settings to help overcome the barriers that this population encounters every day.

## Data Availability

Data sharing not applicable to this article as no datasets were generated or analysed during the current study.

## Abbreviations

CP: Cerebral palsy
ICF: International Classification of Functionning, disability and health
IRT: Item Response Theory
QoL: Quality of Life
SEM: Structural Equation Modelling
SPARCLE: Study of PARticipation of Children with cerebral palsy Living in Europe
WHO: World Health Organisation

## References

[1] Thyen U, Perrin M, Carey WB, Crocker AC, Elias ER, Feldman HM, Coleman WL (2009). Chronic illness. Developmental and behavioral pediatrics.

[2] Hanes JE, Hlyva O, Rosenbaum P, Freeman M, Nguyen T, Palisano RJ (2019). Beyond stereotypes of cerebral palsy: exploring the lived experiences of young Canadians. Child Care Health Dev.

[3] Cussen A, Howie L, Imms C (2012). Looking to the future: adolescents with cerebral palsy talk about their aspirations--a narrative study. Disabil Rehabil.

[4] Jacobson DNO, Löwing K, Hjalmarsson E, Tedroff K (2019). Exploring social participation in young adults with cerebral palsy. J Rehabil Med.

[5] van der Slot WMA, Nieuwenhuijsen C, van den Berg-Emons RJG, Wensink-Boonstra AE, Stam HJ, Roebroeck ME (2010). Participation and health-related quality of life in adults with spastic bilateral cerebral palsy and the role of self-efficacy. J Rehabil Med.

[6] Hilberink SR, Roebroeck ME, Nieuwstraten W, Jalink L, Verheijden JMA, Stam HJ (2007). Health issues in young adults with cerebral palsy: towards a life-span perspective. J Rehabil Med.

[7] Ando N, Ueda S (2000). Functional deterioration in adults with cerebral palsy. Clin Rehabil.

[8] Jacobson DNO, Löwing K, Tedroff K (2020). Health-related quality of life, pain, and fatigue in young adults with cerebral palsy. Dev Med Child Neurol.

[9] McPhee PG, Brunton LK, Timmons BW, Bentley T, Gorter JW (2017). Fatigue and its relationship with physical activity, age, and body composition in adults with cerebral palsy. Dev Med Child Neurol.

[10] Morgan P, McGinley J (2014). Gait function and decline in adults with cerebral palsy: a systematic review. Disabil Rehabil.

[11] McDowell BC, Duffy C, Lundy C (2017). Pain report and musculoskeletal impairment in young people with severe forms of cerebral palsy: a population-based series. Res Dev Disabil.

[12] Bogossian A, Gorter JW, Racine E. Protocol for a scoping review about ethics in transition programmes for adolescents and young adults with neurodisabilities. BMJ Open. 2018;8(8) Available on: https://www.ncbi.nlm.nih.gov/pmc/articles/PMC6104787/. 10.1136/bmjopen-2017-020914 PMC6104787 30121595

[13] Colver A, McConachie H, Le Couteur A, Dovey-Pearce G, Mann KD, McDonagh JE (2018). A longitudinal, observational study of the features of transitional healthcare associated with better outcomes for young people with long-term conditions. BMC Med.

[14] Liptak GS (2008). Health and well being of adults with cerebral palsy. Curr Opin Neurol.

[15] Lyons A, Fletcher G, Farmer J, Kenny A, Bourke L, Carra K (2016). Participation in rural community groups and links with psychological well-being and resilience: a cross-sectional community-based study. BMC Psychol.

[16] Jiang B, Walstab J, Reid SM, Davis E, Reddihough D (2016). Quality of life in young adults with cerebral palsy. Disabil Health J.

[17] Hergenröder H, Blank R (2009). Subjective well-being and satisfaction with life in adults with spastic cerebral palsy: a pilot study of a randomized sample. Dev Med Child Neurol.

[18] Fauconnier J, Dickinson HO, Beckung E, Marcelli M, McManus V, Michelsen SI (2009). Participation in life situations of 8-12 year old children with cerebral palsy: cross sectional European study. BMJ..

[19] Michelsen SI, Flachs EM, Damsgaard MT, Parkes J, Parkinson K, Rapp M (2014). European study of frequency of participation of adolescents with and without cerebral palsy. Eur J Paediatr Neurol EJPN Off J Eur Paediatr Neurol Soc.

[20] Imms C, Adair B (2017). Participation trajectories: impact of school transitions on children and adolescents with cerebral palsy. Dev Med Child Neurol.

[21] Dang VM, Colver A, Dickinson HO, Marcelli M, Michelsen SI, Parkes J (2015). Predictors of participation of adolescents with cerebral palsy: a European multi-Centre longitudinal study. Res Dev Disabil.

[22] Alriksson-Schmidt A, Hägglund G, Rodby-Bousquet E, Westbom L (2014). Follow-up of individuals with cerebral palsy through the transition years and description of adult life: the Swedish experience. J Pediatr Rehabil Med.

[23] Reddihough DS, Jiang B, Lanigan A, Reid SM, Walstab JE, Davis E (2013). Social outcomes of young adults with cerebral palsy. J Intellect Develop Disabil.

[24] Wiegerink DJHG, Roebroeck ME, van der Slot WM, Stam HJ, Cohen-Kettenis PT (2010). South West Netherlands transition research group. Importance of peers and dating in the development of romantic relationships and sexual activity of young adults with cerebral palsy. Dev Med Child Neurol.

[25] Benner JL, Hilberink SR, Veenis T, van der Slot WMA, Roebroeck ME (2017). Course of employment in adults with cerebral palsy over a 14-year period. Dev Med Child Neurol.

[26] Jetha A, Shaw R, Sinden AR, Mahood Q, Gignac MA, McColl MA (2019). Work-focused interventions that promote the labour market transition of young adults with chronic disabling health conditions: a systematic review. Occup Environ Med.

[27] Sentenac M, Ehlinger V, Michelsen SI, Marcelli M, Dickinson HO, Arnaud C (2013). Determinants of inclusive education of 8-12 year-old children with cerebral palsy in 9 European regions. Res Dev Disabil.

[28] Sentenac M, Lach LM, Gariepy G, Elgar FJ (2019). Education disparities in young people with and without neurodisabilities. Dev Med Child Neurol.

[29] Huang I-C, Holzbauer JJ, Lee E-J, Chronister J, Chan F, O’Neil J (2013). Vocational rehabilitation services and employment outcomes for adults with cerebral palsy in the United States. Dev Med Child Neurol.

[30] Benavides FG, Duran X, Gimeno D, Vanroelen C, Martínez JM (2015). Labour market trajectories and early retirement due to permanent disability: a study based on 14 972 new cases in Spain. Eur J Pub Health.

[31] Altwicker-Hámori S, Dratva J (2019). Disability pension receipt in young adults: an analysis of the Swiss social protection and labour market (SESAM) data. BMC Public Health.

[32] Benner JL, Hilberink SR, Veenis T, Stam HJ, van der Slot WM, Roebroeck ME (2017). Long-term deterioration of perceived health and functioning in adults with cerebral palsy. Arch Phys Med Rehabil.

[33] McPhee PG, Benner JL, Balemans ACJ, Verschuren O, van den Berg-Emons RJG, Hurvitz EA (2019). Multimorbidity risk assessment in adolescents and adults with cerebral palsy: a protocol for establishing a core outcome set for clinical research and practice. Trials..

[34] White-Koning M, Grandjean H, Colver A, Arnaud C (2008). Parent and professional reports of the quality of life of children with cerebral palsy and associated intellectual impairment. Dev Med Child Neurol.

[35] Petry K, Maes B, Vlaskamp C (2005). Domains of quality of life of people with profound multiple disabilities: the perspective of parents and direct support staff. J Appl Res Intellect Disabil.

[36] King G, Batorowicz B, Rigby P, Pinto M, Thompson L, Goh F (2014). The leisure activity settings and experiences of youth with severe disabilities. Dev Neurorehabil.

[37] Davey H, Imms C, Fossey E (2015). Our child’s significant disability shapes our lives : experiences of family social participation. Disabil Rehabil.

[38] World Health Organization, éditeur (2001). International classification of functioning, disability and health: ICF.

[39] Colver A, SPARCLE group (2006). Study protocol: SPARCLE--a multi-Centre European study of the relationship of environment to participation and quality of life in children with cerebral palsy. BMC Public Health.

[40] Colver AF, Dickinson HO, SPARCLE Group (2010). Study protocol: determinants of participation and quality of life of adolescents with cerebral palsy: a longitudinal study (SPARCLE2). BMC Public Health.

[41] Colver AF, Dickinson HO, Parkinson K, Arnaud C, Beckung E, Fauconnier J (2011). Access of children with cerebral palsy to the physical, social and attitudinal environment they need: a cross-sectional European study. Disabil Rehabil.

[42] Oskoui M, Coutinho F, Dykeman J, Jetté N, Pringsheim T (2013). An update on the prevalence of cerebral palsy: a systematic review and meta-analysis. Dev Med Child Neurol.

[43] Sellier E, Platt MJ, Andersen GL, Krägeloh-Mann I, De La Cruz J, Cans C (2016). Decreasing prevalence in cerebral palsy: a multi-site European population-based study, 1980 to 2003. Dev Med Child Neurol.

[44] Van Naarden BK, Doernberg N, Schieve L, Christensen D, Goodman A, Yeargin-Allsopp M. Birth prevalence of cerebral palsy: a population-based study. Pediatrics. 2016;137(1):1–9. 10.1542/peds.2015-2872 PMC4703497 26659459

[45] Bax M, Goldstein M, Rosenbaum P, Leviton A, Paneth N, Dan B (2005). Proposed definition and classification of cerebral palsy. Dev Med Child Neurol.

[46] Smithers-Sheedy H, Badawi N, Blair E, Cans C, Himmelmann K, Krägeloh-Mann I (2014). What constitutes cerebral palsy in the twenty-first century?. Dev Med Child Neurol.

[47] Young NL, Rochon TG, McCormick A, Law M, Wedge JH, Fehlings D (2010). The health and quality of life outcomes among youth and young adults with cerebral palsy. Arch Phys Med Rehabil.

[48] Tan SS, van Meeteren J, Ketelaar M, Schuengel C, Reinders-Messelink HA, Raat H (2014). Long-term trajectories of health-related quality of life in individuals with cerebral palsy: a multicenter longitudinal study. Arch Phys Med Rehabil.

[49] Surveillance of Cerebral Palsy in Europe (2000). Surveillance of cerebral palsy in Europe: a collaboration of cerebral palsy surveys and registers. Surveillance of cerebral palsy in Europe (SCPE). Dev Med Child Neurol.

[50] Skevington SM, Lotfy M, O’Connell KA, WHOQOL group (2004). The World Health Organization’s WHOQOL-BREF quality of life assessment: psychometric properties and results of the international field trial. A report from the WHOQOL group. Qual Life Res Int J Qual Life Asp Treat Care Rehab.

[51] Development of the World Health Organization WHOQOL-BREF quality of life assessment. The WHOQOL group. Psychol Med. 1998;28(3):551–8. 10.1017/s0033291798006667. 10.1017/s0033291798006667 9626712

[52] Ravens-Sieberer U, Erhart M, Rajmil L, Herdman M, Auquier P, The European KIDSCREEN Group (2010). Reliability, construct and criterion validity of the KIDSCREEN-10 score: a short measure for children and adolescents’ well-being and health-related quality of life. Qual Life Res.

[53] Ustun TB, Kostanjesek N, Chatterji S, Rehm J, Organization WH. Measuring health and disability : manual for WHO disability assessment schedule (WHODAS 2.0) [internet]: World Health Organization; 2010. Available on: https://apps.who.int/iris/handle/10665/43974

[54] Tuffrey C, Bateman BJ, Colver AC (2013). The questionnaire of Young People’s participation (QYPP): a new measure of participation frequency for disabled young people. Child Care Health Dev.

[55] Dickinson HO, Colver A (2011). Sparcle group. Quantifying the physical, social and attitudinal environment of children with cerebral palsy. Disabil Rehabil.

[56] Schmidt S, Thyen U, Herrmann-Garitz C, Bomba F, Muehlan H (2016). The youth health care measure-satisfaction, utilization, and needs (YHC-SUN)-development of a self-report version of the child health care (CHC-SUN) proxy-measure. BMC Health Serv Res.

[57] Herrmann-Garitz C, Muehlan H, Bomba F, Thyen U, Schmidt S (2017). Konzeption und Erfassung der gesundheitsbezogenen Transitionskompetenz von Jugendlichen mit chronischen Erkrankungen – Entwicklung und Prüfung eines Selbstbeurteilungsinstrumentes. Gesundheitswesen..

[58] McCormick A, Brien M, Plourde J, Wood E, Rosenbaum P, McLean J (2007). Stability of the gross motor function classification system in adults with cerebral palsy. Dev Med Child Neurol.

[59] Elvrum A-KG, Beckung E, Sæther R, Lydersen S, Vik T, Himmelmann K (2017). Bimanual capacity of children with cerebral palsy: intra- and interrater reliability of a revised edition of the bimanual fine motor function classification. Phys Occup Ther Pediatr.

[60] Washington Group Short Set of Disability Questions [Internet]. Washington Group on disability statistics. Available on: http://www.washingtongroup-disability.com/washington-group-question-sets/short-set-of-disability-questions/

[61] Eating and Drinking Ability Classification System (EDACS). Available on: https://www.sussexcommunity.nhs.uk/get-involved/research/chailey-research/eating-drinking-classification.htm

[62] Sellers D, Mandy A, Pennington L, Hankins M, Morris C (2014). Development and reliability of a system to classify the eating and drinking ability of people with cerebral palsy. Dev Med Child Neurol.

[63] Pennington L, Virella D, Mjøen T, da Graça AM, Murray J, Colver A (2013). Development of the Viking speech scale to classify the speech of children with cerebral palsy. Res Dev Disabil.

[64] Hidecker MJC, Paneth N, Rosenbaum PL, Kent RD, Lillie J, Eulenberg JB (2011). Developing and validating the communication function classification system for individuals with cerebral palsy: developing a communication classification system. Dev Med Child Neurol.

[65] Vaizey CJ, Carapeti E, Cahill JA, Kamm MA (1999). Prospective comparison of faecal incontinence grading systems. Gut..

[66] Sandvik H, Espuna M, Hunskaar S (2006). Validity of the incontinence severity index: comparison with pad-weighing tests. Int Urogynecol J Pelvic Floor Dysfunct.

[67] Allen Gomes A, Ruivo Marques D, Meia-Via AM, Meia-Via M, Tavares J, Fernandes da Silva C (2015). Basic scale on insomnia complaints and quality of sleep (BaSIQS): reliability, initial validity and normative scores in higher education students. Chronobiol Int.

[68] Spitzer RL, Kroenke K, Williams JBW, Löwe B (2006). A brief measure for assessing generalized anxiety disorder: the GAD-7. Arch Intern Med.

[69] Kroenke K, Spitzer RL, Williams JB (2001). The PHQ-9: validity of a brief depression severity measure. J Gen Intern Med.

[70] Schwarzer R, Jerusalem M, Weinman J, Wright S, Johnston M (1995). Generalized self-efficacy scale. Measures in health psychology: a user’s portfolio. Causal and control beliefs.

[71] European Social Survey | European Social Survey (ESS). Available on: http://www.europeansocialsurvey.org/

[72] Power MJ, Green AM, The WHOQOL-Dis Group (2010). Development of the WHOQOL disabilities module. Qual Life Res.

[73] Talking Mats | Improving communication, improving lives [Internet]. Talking Mats. Available on: https://www.talkingmats.com/

[74] UNESCO Institute for Statistics (2012). International standard classification of education: ISCED 2011 [internet].

